# Cardiac events after macrolides or fluoroquinolones in patients hospitalized for community-acquired pneumonia: post-hoc analysis of a cluster-randomized trial

**DOI:** 10.1186/s12879-018-3630-7

**Published:** 2019-01-07

**Authors:** Douwe F. Postma, Cristian Spitoni, Cornelis H. van Werkhoven, Leontine J. R. van Elden, Jan Jelrik Oosterheert, Marc J. M. Bonten

**Affiliations:** 1 Julius Center for Health Sciences & Primary Care, University Medical Centre Utrecht, Heidelberglaan 100, 3508 GA Utrecht, the Netherlands; 2 Department of Internal Medicine, Diakonessenhuis Utrecht, Bosboomstraat 1, 3582 KE Utrecht, the Netherlands; 3 Department of Mathematics, Utrecht University, Budapestlaan 6, Room 601, 3584 CD Utrecht, the Netherlands; 4 Department of Internal Medicine & Infectious Diseases, University Medical Centre Utrecht, Heidelberglaan 100, 3508 GA Utrecht, the Netherlands; 5 Department of Pulmonary Medicine, University Medical Centre Utrecht, Heidelberglaan 100, 3508 GA Utrecht, the Netherlands; 6 Department of Medical Microbiology, University Medical Centre Utrecht, Heidelberglaan 100, 3508 GA Utrecht, the Netherlands

**Keywords:** Community-acquired pneumonia, Antibiotics, Macrolides, Fluoroquinolones, Cardiac events, Complications

## Abstract

**Background:**

Guidelines recommend macrolides and fluoroquinolones in patients hospitalized with community-acquired pneumonia (CAP), but their use has been associated with cardiac events. We quantified associations between macrolide and fluoroquinolone use and cardiac events in patients hospitalized with CAP in non-ICU wards.

**Methods:**

This was a post-hoc analysis of a cluster-randomized trial as a cohort study; including patients with a working diagnosis of CAP admitted to non-ICU wards without a cardiac event on admission. We calculated cause-specific hazard ratio’s (HR’s) for effects of time-dependent macrolide and fluoroquinolone exposure as compared to beta-lactam monotherapy on cardiac events, defined as new or worsening heart failure, arrhythmia, or myocardial ischemia during hospitalization.

**Results:**

Cardiac events occurred in 146 (6.9%) of 2107 patients, including heart failure (*n* = 101, 4.8%), arrhythmia (*n* = 53, 2.5%), and myocardial ischemia (*n* = 14, 0.7%). These occurred in 11 of 207 (5.3%), 18 of 250 (7.2%), and 31 of 277 (11.2%) patients exposed to azithromycin, clarithromycin, and erythromycin for at least one day, and in 9 of 234 (3.8%), 5 of 194 (2.6%), and 23 of 566 (4.1%) exposed to ciprofloxacin, levofloxacin, and moxifloxacin, respectively. HR’s for erythromycin, compared to beta-lactam monotherapy, on any cardiac event and heart failure were 1.60 (95% CI 1.09;2.36) and 1.89 (95% CI 1.22;2.91), respectively. HR’s for levofloxacin and moxifloxacin, compared to beta-lactam monotherapy, on any cardiac event were 0.40 (95% CI 0.18;0.87)and 0.56 (95% CI 0.36;0.87), respectively. Findings remained consistent after adjustment for confounders and/or in a sensitivity analysis of radiologically confirmed CAP (*n* = 1604, 76.1%).

**Conclusions:**

Among patients with CAP hospitalized to non-ICU wards, erythromycin use was associated with a 68% increased risk of hospital-acquired cardiac events, mainly heart failure. Levofloxacin and moxifloxacin were associated with a lower risk of heart failure. Although our study does not fully exclude confounding bias, findings remained largely unchanged in crude, adjusted, and sensitivity analyses. These findings may caution the use of erythromycin as empirical therapy in these patients.

**Trial registration:**

The original trial was retrospectively registered under ClinicalTrials.gov Identifier NCT01660204 on August 8th, 2012.

## Background

The use of beta-lactam, macrolide, and fluoroquinolone antibiotics, either alone or in combination, is recommended in international guidelines for empirical treatment of patients hospitalized with community-acquired pneumonia (CAP) [1–3]. However, the evidence-base for the addition of atypical coverage, especially macrolides, to beta-lactam antibiotics in patients with CAP admitted to non-ICU wards has been questioned [4, 5].

Results from observational studies have suggested that the use of macrolides (azithromycin, clarithromycin, and erythromycin) is associated with the occurrence of cardiovascular events, especially in patients with increased cardiovascular risk [6–10]. In a Danish study the relative risk for cardiac events after receiving azithromycin was not increased in the general population [11]. In US patients hospitalized with CAP, azithromycin was associated with a lower risk of all-cause mortality and any cardiac event, although the risk for myocardial infarction up to day 90 after admission did increase (5.1% vs 4.4%; odds ratio 1.17 95% confidence interval (CI) 1.08 to 1.25) with azithromycin use [7]. In another study, clarithromycin use during admission had an increased hazard ratio for cardiovascular events of 1.68 (95% CI 1.18 to 2.38) after one year follow-up among patients hospitalized with CAP [8]. Fluoroquinolones have been associated with an increased risk of arrhythmia in the general population, thus potentially increasing cardiovascular risk when treating CAP patients [10, 12].

Naturally, residual confounding cannot be excluded in these observational studies. However, the possibility of harm caused by macrolides or fluoroquinolones is likely to change the risk-benefit evaluation for atypical coverage in the empirical antibiotic treatment of CAP patients; particularly for macrolides in the context of limited evidence of benefit.

A recent Swiss randomized clinical trial showed a non-significant increase in 30-day mortality of 1.4% (*p* = 0.42) for beta-lactam monotherapy compared to combination therapy with a macrolide in hospitalized CAP patients; clinical stability criteria were met earlier in the combination group (4.5 days) vs beta-lactam monotherapy (5 days) [13]. This is in line with our own cluster-randomized trial, in which we demonstrated that a strategy of beta-lactam monotherapy was non-inferior to beta-lactams combined with a macrolide or fluoroquinolone monotherapy in terms of all-cause mortality for CAP patients admitted to non- intensive care unit (non-ICU) medical wards [14]. Here we present the results of a post-hoc analysis of these data on the association between specific macrolides and fluoroquinolones and the occurrence of cardiac events during hospitalization.

## Methods

### Study setting

We used data from the *Community-Acquired Pneumonia: Study on the initial Treatment with Antibiotics of lower Respiratory Tract infections* (CAP-START) trial, a cluster-randomized cross-over trial comparing three empiric antibiotic strategies for the treatment of CAP. These strategies consisted of empirical treatment with beta-lactam monotherapy, beta-lactam/macrolide combination therapy, or fluoroquinolone monotherapy. The trial was performed in seven Dutch teaching hospitals in the Netherlands from February 2011 until October 2013, during which the strategies rotated in 4-month periods. In this study all patients who were admitted with a working diagnosis of CAP to a non-ICU ward for at least 24 h were eligible for inclusion. Details about the methods and results of the trial have been previously published [14, 15].

For the current analysis we only included patients without evidence of a cardiac event at the time of hospital admission (i.e. in the emergency room).

Patients in the CAP-START trial gave written informed consent within 72 h for data collection. Data collection for the current analysis was approved by the ethics review board of the University Medical Center Utrecht with a waiver for additional informed consent.

### Data collection

Demographic data, co-morbidities, clinical parameters, laboratory data, imaging results, and outcome data were collected from the medical records as part of the CAP-START trial by trained research nurses and were anonymously recorded in case record forms. During the course of the study, four investigators (DFP, CHvW: authors; LAM and KV: see acknowledgements) systematically collected additional data on the presence of cardiac and vascular comorbidities on admission, use of cardiovascular drugs, and the occurrence of cardiac events through chart review.

### Cardiac events

All patients were screened for cardiac events by the same protocol, using pre-specified criteria for new or worsening cardiac events, as described in the literature [16]. We recorded the (co)occurrence of three types of cardiac events: new or worsening arrhythmia, heart failure, and myocardial ischemia. New or worsening arrhythmia was defined as documentation in medical records or electrocardiogram (ECG) of newly recognized atrial fibrillation, flutter, supraventricular tachycardia, ventricular tachycardia or ventricular fibrillation. New or worsening heart failure was defined as clinical signs of new heart failure noted in medical records by treating physicians and a chest X-ray or CT-scan demonstrating evidence of heart failure. New or worsening myocardial ischemia was defined as documentation in medical records of at least two of three criteria: chest pain, acute electrocardiographic changes (ST segment and T wave changes without formation of Q waves, new Q waves or a clear loss of R waves), and/or elevated cardiac enzymes (CPK-MB, troponin-T). Cardiac events were considered worsening when therapeutic action (e.g. increase of diuretic dosage) was needed for already present cardiac disease.

First, admission and discharge letters were examined; if these did not contain detailed information, medical files were reviewed. In case of doubt, findings were presented in a plenary session to obtain group consensus (DFP, CHvW, and JJO: authors; LAM and KV: see acknowledgements) on the occurrence of an event.

### Antibiotic exposure

In the current analyses, macrolide and fluoroquinolone exposure were defined as time-varying exposures starting at the first day of antibiotic prescription until the end of admission. Azithromycin, clarithromycin, and erythromycin were the administered macrolides, and ciprofloxacin, ofloxacin, levofloxacin, and moxifloxacin were the administered fluoroquinolones. Ofloxacin was grouped with and referred to as ciprofloxacin because of the low number of ofloxacin users (*n* = 1).

### Data analysis

Comparisons of baseline variables between groups were analyzed using Chi-squared tests, Fischer’s exact tests, One-way ANOVA’s, and Kruskal-Wallis tests, as appropriate.We assessed the association between macrolide and fluoroquinolone exposure and cardiac events using an extended Cox proportional hazards model with time-varying covariates for exposure to these antibiotics. Antibiotic exposure was modelled as present from the starting date of the antibiotic prescription until the end of admission, as previously mentioned. This ensured that cardiac events occurring before an antibiotic was started could not be attributed to this antibiotic. Crude models included all six antibiotics (azithromycin, clarithromycin, erythromycin, ciprofloxacin, levofloxacin, and moxifloxacin) as time-dependent covariates and the different cardiac events (any cardiac event, heart failure, and arrhythmia) as outcomes. The calculated hazard ratios for time-dependent exposure to macrolides or quinolones are in comparison to patients who did not receive macrolide or fluoroquinolone antibiotics at any time during admission i.e. who received beta-lactam monotherapy. Hospital discharge, transfer or death during admission led to right censoring in the analysis.

To calculate adjusted hazard ratios, we added the following confounders to our models: PSI score, number of other cardiac comorbidities, statin use, anti-platelet use, smoking history, positive pneumococcal urinary antigen test, and serum C-reactive protein. The linearity of continuous variables was checked visually by plotting martingale residuals.

To assess the effect of the domain definition of clinical versus radiologically proven CAP, we repeated all analyses in the subgroup of patients with radiologically proven CAP.

As an additional sensitivity analysis we performed a competing risk analysis, using three competing outcomes during admission: a new or worsening cardiac event, in-hospital death, or hospital discharge. Following competing risk methodology, we set out to calculate cause-specific hazard ratios and sub-distribution hazard ratios for our exposures of interest for each outcome [17]. The cause-specific hazard ratio is an estimate of the instantaneous risk of failing from a certain outcome, given that no outcome has yet occurred. Patients failing from competing outcomes are censored in these analyses. The cause-specific hazard ratio is equal to our extended Cox proportional hazards model mentioned above. The sub-distribution hazard ratio (SDHR) is an estimate of failing from an outcome of interest when competing outcomes have or have not occurred; thus it describes the ‘net effect’ a variable has on the cumulative incidence function [18].

Missing data were imputed by multiple imputation, except for data on respiratory rate, heart rate, and confusion at admission; the values for these variables were assumed to be normal when not explicitly documented in the medical records as they are frequently not recorded if within the normal range. *P*-values below .05 were considered statistically significant. All analyses were performed in R software, version 3.2.0 [19].

### Role of the funding source

The original trial was supported by a grant from The Netherlands Organization for Health Research and Development. This source did not have a role in the design, conduct and reporting of the study.

## Results

### Patients

**Fig. 1:**
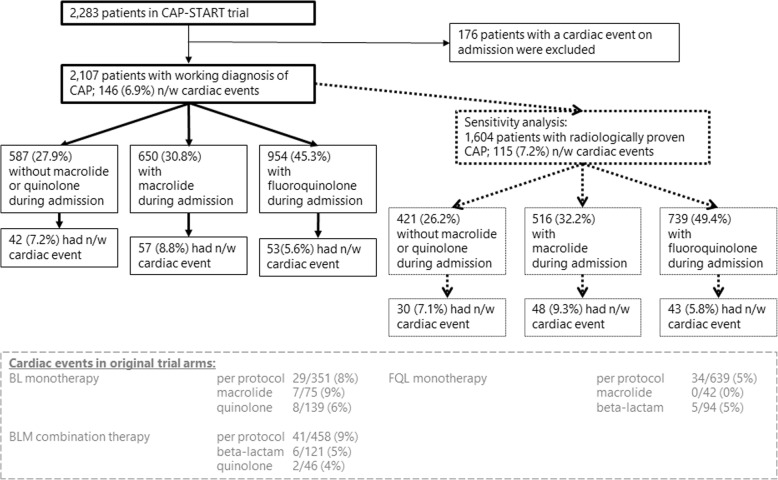
Inclusion of patients and cardiac event rates per population. n/w new or worsening; BL beta-lactam; BLM beta-lactam combination with macrolide; FQL fluoroquinolone. The population for the primary analysis consisted of patients with a working diagnosis of CAP; the population for the sensitivity analysis consisted of the patients that developed radiological evidence of CAP during the first 48 h of admission. Crude new or worsening cardiac event rates are presented from the original trial arms for patients only receiving the cluster-randomized antibiotic strategy (per protocol) or who were non-adherent to the strategy

We included 2107 patients who were admitted to non-ICU wards with a working diagnosis of CAP and without a cardiac event on admission (Fig. 1). Baseline characteristics and outcomes, stratified by receipt of any macrolide or fluoroquinolone during admission, are displayed in Table 1. The median length of stay was 6 days (IQR 4–9 days). There were 146 (6.9%) patients with new cardiac events and 66 (3.1%) died during hospital stay (Table 1, Fig. 1). The self-reported use of antibiotics before admission occurred in just over 30% of patients, with a median length of 3 days (IQR 1 to 6 days); 5.4% of patients reported the use of macrolides and 2.5% reported fluoroquinolones before admission (Table 1). The microbial etiology, stratified by the occurrence of any cardiac event, is presented in Table 2.

**Table 1:** Baseline patient characteristics and outcomes stratified by use of any macrolide or fluoroquinolone during admission

	All patients	Use of any macrolide during admission	Use of any fluoroquinoloneduring admission	No macrolide or fluoroquinolone during admission	*P* value
*n*	2107	650	954	587	
Age	69 (58;79)	69 (57;78)	69 (58;79)	71 (60.5;79)	0.609
Male sex	1217 (57.8%)	388 (59.7%)	556 (58.3%)	332 (56.6%)	0.949
Nursing home residence^1^	100 (4.8%)	27 (4.2%)	48 (5.1%)	31 (5.4%)	0.684
Radiologically proven CAP	1604 (76.1%)	516 (79.4%)	739 (77.5%)	421 (71.7%)	0.684
Smoking current/ever	1599 (77.9%)	491 (77.1%)	729 (78.2%)	449 (79.2%)	0.998
Co-morbidities					
History of cardiac disease	733 (34.8%)	193 (29.7%)	342 (35.8%)	227 (38.7%)	0.122
History of ischemic heart disease	415 (19.7%)	102 (15.7%)	190 (19.9%)	138 (23.5%)	0.044
History of atrial fibrillation	299 (14.2%)	88 (13.5%)	141 (14.8%)	86 (14.7%)	0.935
History of heart failure	198 (9.4%)	58 (8.9%)	91 (9.5%)	62 (10.6%)	0.835
History of vascular disease*	458 (21.7%)	130 (20.0%)	219 (23.0%)	133 (22.7%)	0.698
History of hypertension	643 (30.5%)	211 (32.5%)	285 (29.9%)	178 (30.3%)	0.874
History of COPD	785 (37.3%)	231 (35.5%)	352 (36.9%)	232 (39.5%)	0.805
History of diabetes	339 (16.1%)	83 (12.8%)	175 (18.3%)	97 (16.5%)	0.088
Medication use^2^					
Antibiotic use before admission^3^	702 (34.1%)	215 (33.9%)	345 (36.7%)	178 (31.3%)	0.410
Beta-lactam	376 (18.3%)	112 (17.7%)	192 (20.4%)	87 (15.3%)	0.168
Macrolide	111 (5.4%)	42 (6.6%)	52 (5.5%)	29 (5.1%)	0.674
Fluoroquinolone	52 (2.5%)	15 (2.4%)	28 (3.0%)	13 (2.3%)	0.805
Other antibiotic	238 (11.6%)	65 (10.3%)	114 (12.1%)	71 (12.5%)	0.687
Use of antiplatelet agents	575 (27.4%)	186 (28.8%)	252 (26.5%)	170 (29.1%)	0.818
Use of anticoagulants	345 (16.4%)	85 (13.2%)	165 (17.4%)	104 (17.8%)	0.188
Use of antihypertensives	1057 (50.4%)	299 (46.3%)	485 (51.1%)	314 (53.7%)	0.486
Use of statins	629 (30.0%)	185 (28.6%)	285 (30.0%)	186 (31.8%)	0.843
Severity scores					
PSI score	83.7 ± 28.4	82.0 ± 27.9	84.6 ± 28.7	85.2 ± 28.4	0.007
CURB65 score§	1 (1;2)	1 (0;2)	1 (1;2)	1 (1;2)	0.042
Outcomes					
n/w cardiac event	146 (6.9%)	57 (8.8%)	53 (5.6%)	42 (7.2%)	0.144
n/w arrhythmia	53 (2.5%)	19 (2.9%)	21 (2.2%)	14 (2.4%)	0.845
n/w heart failure	101 (4.8%)	46 (7.1%)	34 (3.6%)	27 (4.6%)	0.024
n/w myocardial ischemia	14 (0.7%)	2 (0.3%)	7 (0.7%)	6 (1.0%)	0.486
Transfer to other hospital	12 (0.6%)	1 (0.2%)	7 (0.7%)	5 (0.9%)	0.320
In-hospital mortality	66 (3.1%)	27 (4.2%)	35 (3.7%)	13 (2.2%)	0.262

Values are medians (interquartile range) unless otherwise noted. Plus-minus values are means ± SD

COPD denotes chronic obstructive pulmonary disease

*includes cerebrovascular, peripheral artery, and thrombo-embolic disease

¶ The PSI score uses 20 clinical measures to predict risk of death within 30 days, with results ranging from 0.1% (in patients with a score of 0–50) to 27.0% (in patients with a score > 131)

§ The CURB-65 score is calculated by assigning 1 point each for confusion, uremia (blood urea nitrogen ≥ 20 mg per deciliter), high respiratory rate (≥30 breaths per minute), low systolic blood pressure (< 90 mmHg) or diastolic blood pressure (≤60 mmHg), and an age of 65 years or older, with a higher score indicating a higher risk of death within 30 days

^1^Between 1 and 2.2% missing values for each group

^2^Between 0.3–0.6% missing values for each group

^3^Self-reported use of antibiotics before admission

**Table 2:** Microbial aetiology of CAP stratified by cardiac event

	No cardiac event		Any cardiac event	
	Proven	Possible	Proven	Possible
*Streptococcus pneumoniae*	191 (12.8%)	48 (3.2%)	20 (17.4%)	1 (0.9%)
*Haemophilus influenzae*	5 (0.3%)	102 (6.9%)	1 (0.9%)	9 (7.8%)
*Moraxella catarrhalis*	–	16 (1.1%)	–	2 (1.7%)
*Staphylococcus aureus*	7 (0.5%)	41 (2.8%)	–	4 (3.5%)
Other gram positives	8 (0.5%)	11 (0.7%)	1 (0.9%)	–
*Escherichia coli*	7 (0.5%)	33 (2.2%)	2 (1.7%)	2 (1.7%)
*Klebsiella pneumoniae*	1 (0.1%)	10 (0.7%)	–	2 (1.7%)
*Pseudomonas aeruginosa*	–	29 (1.9%)	–	2 (1.7%)
Other gram negatives	5 (0.3%)	56 (3.8%)	–	6 (5.2%)
*Legionella pneumophila*	14 (0.9%)	1 (0.1%)	–	–
*Mycoplasma pneumoniae*	–	21 (1.4%)	–	–
Mycobacteria	–	2 (0.1%)	–	–
Viruses	–	40 (2.7%)	–	1 (0.9%)
Fungi / yeast	–	30 (2.0%)	1 (0.9%)	1 (0.9%)
No Pathogen	–	944 (63.4%)	–	74 (64.3%)

Proven pathogens: based on pathogens detected in blood cultures, pleural fluid cultures, and urinary antigen tests (BINAX Now for *S. pneumoniae* and *L. pneumophila*). Possible pathogens: based on pathogens detected in sputum cultures, broncho-alveolar lavage fluid cultures, and serology. Candida species cultured from sputum and common skin contaminants from blood cultures where antibiotic treatment was not changed, were considered as contamination

Six hundred fifty patients (30.8%) received at least one day of macrolide treatment, and 954 (45.3%) received at least one day of fluoroquinolones during admission. Treatment mostly started on the first day of admission (Table 3). Erythromycin was predominantly (94.9%) administered intravenously, whereas other macrolides were only used orally (as they are not available for intravenous administration in the Netherlands). Azithromycin and clarithromycin were predominantly prescribed together with ceftriaxone or amoxicillin with or without clavulanic acid, while erythromycin was predominantly prescribed with penicillin or cefuroxime. Levofloxacin and moxifloxacin were usually prescribed as monotherapy, while ciprofloxacin was administered together with beta-lactams (Table 4). The proportion of patients receiving fluoroquinolones that started with oral treatment on admission ranged from 30.4% for ciprofloxacin to 67.5% for moxifloxacin (Table 3). Patients receiving macrolides, as compared to the overall cohort, were less likely to have cardiac comorbidities (29.7% vs 34.8%, *p*-value 0.122), a history of diabetes (12.8% vs 16.1%, *p*-value 0.088), and anticoagulants (13.2% vs 16.4%, *p*-value 0.188). There were no obvious differences in baseline characteristics such as co-morbidities or use of cardiovascular medication for patients receiving fluoroquinolones as compared to the overall cohort. The disease severity upon admission, as represented by the PSI score or CURB-65 score, was slightly, but significantly, lower in patients using any macrolide during admission (PSI score 82 vs 85, p-value 0.007). (Table 1). The 146 patients with cardiac events developed 101 (4.8%) first episodes of heart failure, 53 (2.5%) of arrhythmia, and 14 (0.7%) of myocardial ischemia.

**Table 3:** Starting days and crude event rates for different macrolides and fluoroquinolones

Macrolides		Azithromycin	Clarithromycin	Erythromycin
Patients with antibiotic any time during admission		207	250	277
Starting day of antibiotic during admission¶		1 (0–2)	0 (0–1)	0 (0–0)
Percentage starting antibiotic intravenously §		–	–	263 (94.9%)
Cardiac event	a) Any type	11 (5.3%)	18 (7.2%)	31 (11.2%)
	b) Heart failure	9 (4.3%)	14 (5.6%)	26 (9.4%)
	c) Arrhythmia	6 (2.9%)	5 (2%)	10 (3.6%)
Fluoroquinolones		Ciprofloxacin	Levofloxacin	Moxifloxacin
Patients with antibiotic any time during admission		234*	194	566
Starting day of antibiotic during admission¶		1 (0–3)	0 (0–0)	0 (0–0)
Percentage starting antibiotic intravenously		76 (32.5%)	111 (57.2%)	394 (69.6%)
Cardiac event	a) Any type	9 (3.8%)	5 (2.6%)	23 (4.1%)
	b) Heart failure	9 (3.8%)	3 (1.5%)	16 (2.8%)
	c) Arrhythmia	5 (2.1%)	3 (1.5%)	11 (1.9%)

Values are numbers (percentages) unless otherwise noted. ¶Median (interquartile range) *One patient received ofloxacin

§ Azithromycin and clarithromycin are not available for intravenous administration in the Netherlands

**Table 4:** Administered beta-lactams at admission for patients with or without different macrolides and fluoroquinolones

	Azithromycin (*N* = 151)	Clarithromycin (*N* = 222)	Erythromycin (*N* = 277)	Ciprofloxacin (*N* = 206)	Levofloxacin (*N* = 194)	Moxifloxacin (*N* = 554)	No macrolides or fluoroquinolones (*N* = 587)
Amoxicillin	17 (11.0%)	53 (22.3%)	25 (8.4%)	70 (34.0%)	2 (1.0%)	16 (2.9%)	149 (25.4%)
Amoxicillin/clavulanic acid	37 (23.9%)	148 (62.2%)	33 (11.1%)	69 (33.5%)	6 (3.1%)	18 (3.2%)	291 (49.6%)
Ceftriaxone	72 (46.5%)	17 (7.1%)	31 (10.4%)	30 (14.6%)	4 (2.1%)	28 (5.1%)	95 (16.2%)
Cefuroxime	5 (3.2%)	12 (5.0%)	96 (32.2%)	10 (4.9%)	0 (0.0%)	3 (0.5%)	24 (4.1%)
Cefotaxime	0 (0.0%)	1 (0.4%)	3 (1.0%)	1 (0.5%)	0 (0.0%)	0 (0.0%)	1 (0.2%)
Ceftazidime	4 (2.6%)	2 (0.8%)	3 (1.0%)	8 (3.9%)	1 (0.5%)	1 (0.2%)	12 (2.0%)
Penicillin	5 (3.2%)	10 (4.2%)	146 (49.0%)	20 (9.7%)	8 (4.1%)	10 (1.8%)	9 (1.5%)

Values are numbers (column percentages) unless otherwise noted

### Macrolide exposure and cardiac events

In the patients receiving macrolides (*n* = 650), 207, 250, and 277 patients were exposed to azithromycin, clarithromycin, and erythromycin for at least one day, of which 11 (5.3%), 18 (7.2%), and 31 (11.2%) developed a cardiac event, respectively. The crude hazard ratio for a cardiac event after erythromycin use was 1.60 (95% CI 1.09; 2.36), and was 1.89 (95% CI 1.22; 2.91) for heart failure specifically. After adjustment for confounders hazard ratios were 1.68 (95% CI 1.07; 2.62) for any cardiac event and 2.08 (95% CI 1.25; 3.46) for heart failure. Adjusted hazard ratios for any cardiac event were 0.89 (95% CI 0.48; 1.67) and 1.06 (95% CI 0.61; 1.83) for azithromycin and clarithromycin, respectively. The numbers of cardiac events due to arrhythmia were too low for meaningful interpretations.(Table 5).

**Table 5:** Hazard ratio’s for macrolides during admission

	Working diagnosis of CAP *N* = 2107; 146 n/w cardiac events		Radiologically proven CAP *N* = 1604; 115 n/w cardiac events	
Outcome *antibiotic*	Crude HR (CI)	Adjusted HR (CI)	Crude HR (CI)	Adjusted HR (CI)
n/w Cardiac event				
*azithromycin*	0.70 (0.39;1.26)	0.76 (0.42;1.35)	0.59 (0.29;1.18)	0.66 (0.33;1.32)
*clarithromycin*	0.84 (0.51;1.38)	1.03 (0.62;1.70)	0.88 (0.52;1.51)	1.09 (0.63;1.88)
*erythromycin*	1.60 (1.09;2.36)	1.82 (1.23;2.68)	1.53 (1.00;2.35)	1.67 (1.09;2.57)
n/w Heart failure				
*azithromycin*	0.73 (0.38;1.41)	0.78 (0.40;1.52)	0.61 (0.28;1.35)	0.69 (0.32;1.53)
*clarithromycin*	0.93 (0.53;1.64)	1.17 (0.66;2.08)	0.92 (0.49;1.70)	1.20 (0.64;2.24)
*erythromycin*	1.89 (1.22;2.91)	2.11 (1.36;3.26)	1.67 (1.03;2.71)	1.77 (1.09;2.87)
n/w Arrhythmia				
*azithromycin*	1.00 (0.42;2.39)	1.03 (0.43;2.47)	0.77 (0.27;2.21)	0.85 (0.30;2.45)
*clarithromycin*	0.76 (0.31;1.84)	0.87 (0.36;2.12)	0.88 (0.36;2.17)	1.02 (0.41;2.53)
*erythromycin*	1.25 (0.62;2.49)	1.28 (0.64;2.57)	1.30 (0.62;2.72)	1.32 (0.63;2.78)

n/w = new or worsening

Hazard ratio’s (HR’s) with 95% confidence intervals (CIs) from Cox PH models for time-dependent exposure to different macrolides during admission on cardiac events. Adjusted HR’s are adjusted for confounders mentioned in Methods

**Fig. 2:**
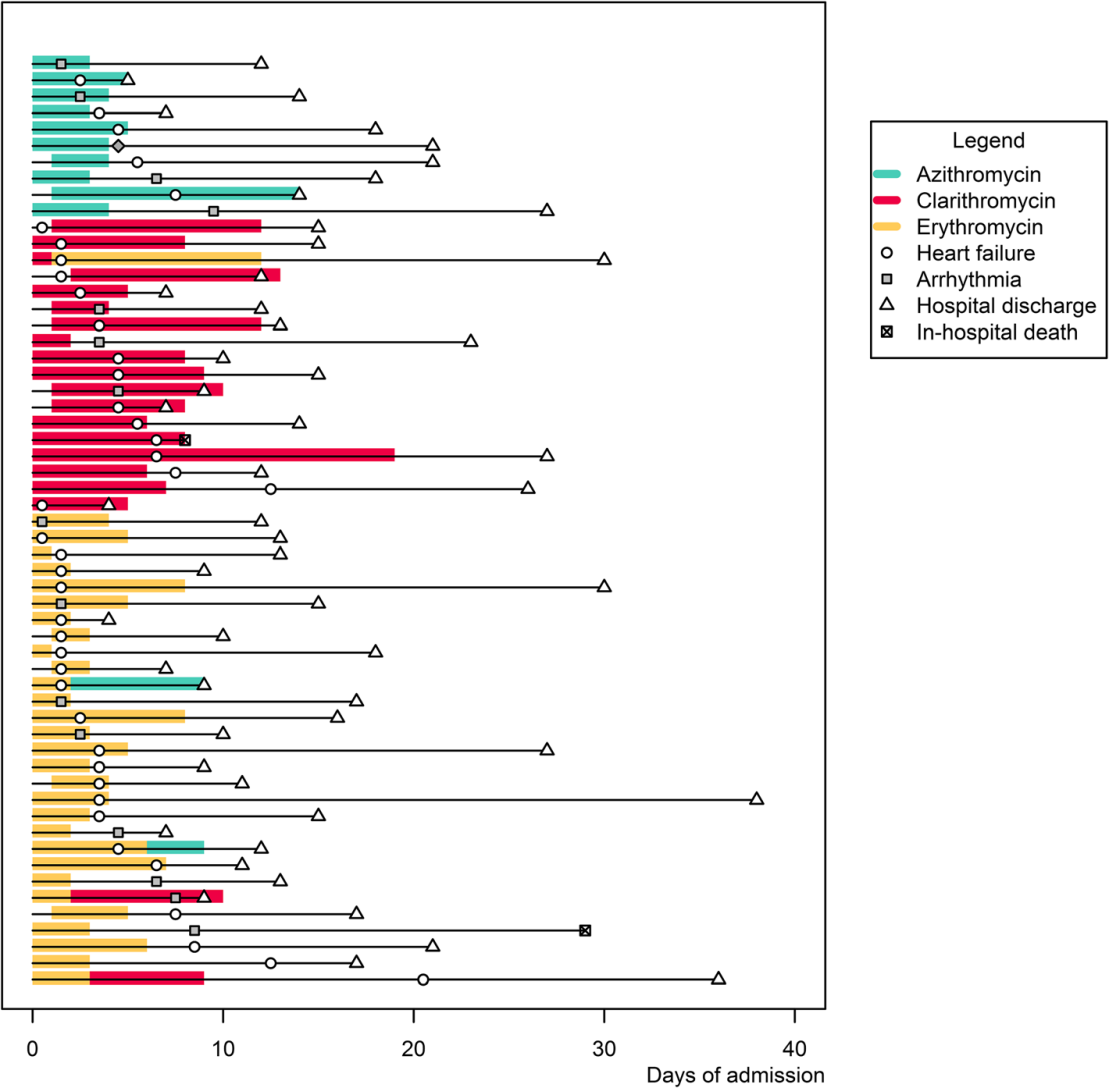
Timing of events in macrolide users with cardiac events. Figure depicts timing of events through admission for each patient which used a macrolide and experienced a cardiac event. Individual patients are on the y-axis and time in days of admission is on the x-axis. Cardiac events occurring before prescription of a macrolide were not attributed to the antibiotic in statistical models as antibiotics were modelled time-dependently

Risk estimates were similar in the subgroup analysis of patients with radiologically confirmed CAP. (Table 5) In most macrolide-exposed patients that developed cardiac events (*n* = 45, 78.9%), macrolides were started on the day of admission. (Fig. 2).

### Time-dependent exposure to fluoroquinolones

In the fluoroquinolone group (*n* = 954), 234, 194, and 566 patients were exposed to ciprofloxacin, levofloxacin, and moxifloxacin, of which 9 (3.8%), 5 (2.6%), and 23 (4.1%) developed a cardiac event, respectively. (Table 3) Both levofloxacin and moxifloxacin were associated with lower risks of any cardiac event in crude analyses, with a hazard ratio of 0.40 (95% CI 0.18; 0.87) for levofloxacin and 0.56 (95% CI 0.36; 0.87) for moxifloxacin. Hazard ratios for heart failure specifically were 0.25 (95% CI 0.08; 0.80) for levofloxacin and 0.48 (95% CI 0.27; 0.84) for moxifloxacin. Associations for any cardiac event remained comparable after adjustment for confounders. The association between moxifloxacin and heart failure lost statistical significance after adjustment (Table 6). The numbers of cardiac events due to arrhythmia were too low for meaningful interpretations. Ciprofloxacin was not associated with a significantly changed hazard ratio for cardiac events. (Table 6).

**Table 6:** Hazard ratio’s for fluoroquinolones during admission

	Working diagnosis of CAP *N* = 2107; 146 n/w cardiac events		Radiologically proven CAP *N* = 1604; 115 n/w cardiac events	
Outcome *antibiotic*	Crude HR (CI)	Adjusted HR (CI)	Crude HR (CI)	Adjusted HR (CI)
n/w cardiac event				
*ciprofloxacin*	0.77 (0.43;1.37)	0.70 (0.39;1.26)	0.81 (0.44;1.50)	0.72 (0.39;1.33)
*levofloxacin*	0.40 (0.18;0.87)	0.43 (0.20;0.93)	0.33 (0.12;0.91)	0.36 (0.13;0.98)
*moxifloxacin*	0.56 (0.36;0.87)	0.56 (0.36;0.88)	0.53 (0.32;0.86)	0.54 (0.33;0.89)
n/w Heart failure				
*ciprofloxacin*	0.71 (0.36;1.44)	0.65 (0.32;1.31)	0.71 (0.34;1.50)	0.62 (0.29;1.31)
*levofloxacin*	0.25 (0.08;0.80)	0.27 (0.08;0.86)	0.12 (0.02;0.85)	0.12 (0.02;0.88)
*moxifloxacin*	0.48 (0.27;0.84)	0.50 (0.28;0.87)	0.43 (0.23;0.81)	0.46 (0.24;0.85)
n/w Arrhythmia				
*ciprofloxacin*	0.83 (0.32;2.12)	0.75 (0.29;1.93)	0.73 (0.25;2.09)	0.64 (0.22;1.86)
*levofloxacin*	0.50 (0.15;1.64)	0.49 (0.15;1.62)	0.71 (0.21;2.39)	0.70 (0.21;2.35)
*moxifloxacin*	0.66 (0.33;1.34)	0.66 (0.33;1.34)	0.69 (0.32;1.46)	0.71 (0.33;1.51)

n/w = new or worsening

Hazard ratio’s (HR’s) with 95% confidence intervals (CIs) from Cox PH models for time-dependent exposure to different fluoroquinolones during admission on cardiac events. Adjusted HR’s are adjusted for confounders mentioned in Methods

### Competing risk analysis

The calculated sub-distribution hazard ratios from our competing risk analysis were comparable to the primary analysis for all associations. (Tables 7 & 8).

**Table 7:** Sub-distribution hazard ratio’s for macrolides during admission

	Working diagnosis of CAP *N* = 2107; 146 n/w cardiac events		Radiologically proven CAP *N* = 1604; 115 n/w cardiac events	
Outcome *antibiotic*	Crude HR (CI)	Adjusted HR (CI)	Crude HR (CI)	Adjusted HR (CI)
n/w Cardiac event				
*azithromycin*	0.86 (0.48;1.53)	0.93 (0.52;1.67)	0.73 (0.37;1.46)	0.84 (0.42;1.68)
*clarithromycin*	1.11 (0.68;1.83)	1.34 (0.81;2.22)	1.19 (0.70;2.04)	1.47 (0.85;2.52)
*erythromycin*	1.73 (1.17;2.55)	1.99 (1.35;2.94)	1.66 (1.08;2.56)	1.87 (1.21;2.88)
n/w Heart failure				
*azithromycin*	0.90 (0.46;1.74)	1.00 (0.51;1.94)	0.76 (0.35;1.67)	0.91 (0.41;2.00)
*clarithromycin*	1.25 (0.71;2.20)	1.57 (0.89;2.79)	1.26 (0.68;2.33)	1.67 (0.89;3.12)
*erythromycin*	2.06 (1.33;3.18)	2.35 (1.52;3.65)	1.84 (1.13;3.00)	2.01 (1.23;3.28)
n/w Arrhythmia				
*azithromycin*	1.22 (0.51;2.91)	1.28 (0.54;3.07)	0.96 (0.33;2.73)	1.09 (0.38;3.14)
*clarithromycin*	1.02 (0.42;2.47)	1.18 (0.48;2.85)	1.21 (0.49;2.98)	1.43 (0.57;3.55)
*erythromycin*	1.37 (0.69;2.74)	1.44 (0.72;2.88)	1.44 (0.68;3.02)	1.49 (0.71;3.15)

n/w = new or worsening

Sub-distribution hazard ratio’s (SDHR’s) with 95% confidence intervals (CIs) from Cox PH models for time-dependent exposure to different macrolides during admission on cardiac events. Adjusted SDHR’s are adjusted for confounders mentioned in Methods

**Table 8:** Sub-distribution hazard ratio’s for fluoroquinolones during admission

	Working diagnosis of CAP *N* = 2107; 146 n/w cardiac events		Radiologically proven CAP *N* = 1604; 115 n/w cardiac events	
Outcome *antibiotic*	Crude HR (CI)	Adjusted HR (CI)	Crude HR (CI)	Adjusted HR (CI)
n/w cardiac event				
*ciprofloxacin*	1.09 (0.61;1.94)	0.95 (0.53;1.70)	1.18 (0.64;2.17)	1.00 (0.54;1.85)
*levofloxacin*	0.49 (0.23;1.07)	0.52 (0.24;1.13)	0.40 (0.15;1.11)	0.43 (0.15;1.18)
*moxifloxacin*	0.68 (0.44;1.06)	0.68 (0.43;1.06)	0.65 (0.40;1.07)	0.66 (0.40;1.10)
n/w Heart failure				
*ciprofloxacin*	1.05 (0.52;2.10)	0.91 (0.46;1.84)	1.06 (0.51;2.24)	0.89 (0.42;1.87)
*levofloxacin*	0.31 (0.10;0.98)	0.33 (0.10;1.05)	0.14 (0.02;1.02)	0.15 (0.02;1.08)
*moxifloxacin*	0.58 (0.33;1.01)	0.60 (0.35;1.06)	0.53 (0.29;1.00)	0.57 (0.30;1.06)
n/w Arrhythmia				
*ciprofloxacin*	1.19 (0.46;3.04)	1.04 (0.40;2.66)	1.08 (0.38;3.10)	0.93 (0.32;2.66)
*levofloxacin*	0.62 (0.19;2.05)	0.61 (0.19;2.02)	0.88 (0.26;2.95)	0.87 (0.26;2.91)
*moxifloxacin*	0.81 (0.40;1.65)	0.82 (0.40;1.65)	0.86 (0.40;1.83)	0.89 (0.42;1.89)

n/w = new or worsening

Sub-distribution hazard ratio’s (SDHR’s) with 95% confidence intervals (CIs) from Cox PH models for time-dependent exposure to different fluoroquinolones during admission on cardiac events. Adjusted SDHR’s are adjusted for confounders mentioned in Methods

## Discussion

In this study of 2107 patients hospitalized with CAP to non-ICU wards, of which 146 (6.9%) developed a cardiac event during admission, erythromycin use was associated with a 68% higher hazard of cardiac events during hospital admission. This effect almost completely resulted from episodes of new or worsening heart failure. In contrast, levofloxacin and moxifloxacin were associated with a lower risk of cardiac events, mainly because of a lower risk of heart failure during admission.

This was a post-hoc analysis of a cluster-randomized cross-over study evaluating three antibiotic treatment strategies for patients hospitalized with CAP [14]. Baseline differences between patients who did or did not receive macrolides or fluoroquinolones during admission were small. Disease severity scores (i.e. PSI and CURB-65) at baseline were slightly lower in patient who received macrolides at any time during admission, although these differences do not seem clinically significant. If anything, classical cardiovascular risk factors were less prevalent in patients that received macrolides such as erythromycin. Hence, it is possible that physicians considered these antibiotics as potential risks for cardiac events in CAP patients, which may have attenuated the crude effect estimates in this analysis [20]. Consequently, the effect estimate between macrolides and cardiac events increased after adjustment for potential confounders. The findings were largely unchanged in patients with radiologically confirmed CAP. As associations between erythromycin use and competing outcomes such as in-hospital death or hospital discharge could change the interpretation of effect estimates, we performed a competing risk analysis which did not change our results [17]. Assessing associations with the original trial arms would have diluted effect sizes too much due to protocol non-adherence, while using subgroups (per-protocol and non-adherent populations) would have led to the same loss of randomization, but with smaller groups, as our current observational cohort study approach. We have tried to optimally correct for indication bias by using time-varying effects for antibiotic exposure and adjustment for confounders.

Several biological mechanisms could explain the observed associations between erythromycin, levofloxacin, and moxifloxacin use and cardiac events, which could be attributed almost completely to occurrences of heart failure. The proposed explanation for this is the volume and sodium load associated with intravenous administration of erythromycin, compared to regimens including either other macrolides (which are only orally available) or monotherapy with levofloxacin or moxifloxacin. The latter fluoroquinolones are also frequently given as oral treatment as the bioavailability of these antibiotics is known to be high [21]. When looking at the different beta-lactams co-administered with the three types of macrolides, erythromycin was most frequently prescribed together with penicillin. Still, we assume that different beta-lactams are not cardiotoxic and that the sodium and volume load does not differ too much between beta-lactams (Table 4). Unfortunately, we did not have data on fluid balances (intravenous fluid administration) for our patients to further strengthen this hypothesis. In addition, erythromycin is a pro-arrhythmic drug, metabolized through CYP3A4 thus frequently interacting with other drugs, which can prolong the QT-interval [22, 23]. Such effects would most likely occur shortly after start of treatment, and can lead to anything from self-limiting episodes of arrhythmia to severe arrhythmia or Torsade des Pointes, and consequently to demand ischemia and/or decompensated heart failure. Yet, the current analysis might have been underpowered to identify associations between use of any macrolide and occurrence of arrhythmias, as compared to previous analyses in out-patient populations [6, 9, 10, 12]. Finally, erythromycin might have a weaker anti-inflammatory effect than other macrolides, thus leading to more heart failure if this was predominantly driven by pro-inflammatory cytokines [24, 25].

Our findings do not support previous findings of increased risks for cardiac events when using azithromycin or clarithromycin in hospitalized CAP patients [7, 8]. In these studies all hospitalized patients with CAP, including those needing ICU-admission, were included with considerable longer follow-up periods for cardiac events, ranging from 90 days after discharge to one year after admission. In both studies, cardiac events mainly involved myocardial ischemia, which was a relatively infrequent event during hospitalization in our study. Moreover, fluoroquinolones have been associated with an increased risk for arrhythmia in the general population, [10, 12] as mentioned before, our study was probably underpowered to properly assess this association.

Our post-hoc analysis has limitations. The occurrence of cardiac events during the course of the study was based on medical chart review in an open-label trial. This methodology has a risk for misclassification and observational bias. Random misclassification might have led to general underreporting of cardiac events, which would affect the precision of the effect estimates. More importantly, observational bias might have occurred if treating physicians would be more alert to report cardiac events in medical charts of erythromycin users, which would have biased the results from null. As this could also apply to registration of cardiac events by researchers, we used pre-specified criteria for recording cardiac events to reduce this bias. Unfortunately, we were not able to systematically gather information on the etiology of heart failure, e.g. true left-ventricular heart failure as compared to sepsis-induced episodes, which would have helped in identifying causal mechanisms. Another limitation could be residual confounding by indication. Although patient groups were comparable in terms of disease severity at baseline (Table 1), clinical stability during the first two to four days of admission is an important prognostic factor for adverse outcomes in CAP [26, 27]. Therefore, clinical deterioration during the first days of admission might have stimulated treating physicians to add macrolides or fluoroquinolones, creating confounding by indication. Since changes in disease severity during these first days of admission were not determined, this could not be accounted for. Still, the majority of erythromycin users with a cardiac event (*n* = 25, 80.6%) started with erythromycin on the day of admission. (Fig. 2) Lastly, we did not have data on concomitant use of drugs interacting with macrolides or fluoroquinolones, nor did we determine specific causes of death. The latter could have increased the efficacy of end-point detection, and might have allowed discrimination between sudden cardiac death, cardiovascular death, and death due to other causes, although cause-of-death statements are not always interpreted unequivocally [28].

## Conclusion

In this post-hoc analysis, intravenous erythromycin use, but not oral azithromycin or clarithromycin use, increases the risk for cardiac events, especially heart failure, in patients hospitalized with CAP to non-ICU wards. Volume overload associated with intravenous administration of erythromycin in elderly could be one of the mechanisms for heart failure. Inversely, this might explain why levofloxacin and moxifloxacin were associated with a lower risk of cardiac events, mainly heart failure, during admission. Although our study does not fully exclude confounding bias, findings remained largely unchanged in crude, adjusted, and sensitivity analyses. Together with the absence of survival benefit these findings may caution the use of intravenous erythromycin for empirical treatment of CAP patients admitted to non-ICU wards.

## Abbreviations

CAP: Community-acquired pneumonia
ECG: Electrocardiogram
ICU: Intensive Care Unit
